# The Effect of Prednisone on Tuberculin Skin Test Reaction in Patients with Rheumatoid Arthritis

**DOI:** 10.1155/2018/2586916

**Published:** 2018-10-21

**Authors:** Olga Reitblat, Tsahi T. Lerman, Ornit Cohen, Tatiana Reitblat

**Affiliations:** ^1^Barzilai Medical Center, Ashkelon, Israel; ^2^Ben Gurion University of the Negev, Beer-Sheva, Israel; ^3^Rheumatology Unit, Barzilai Medical Center, Ashkelon, Israel

## Abstract

**Objectives:**

To assess the correlation between prednisone and methotrexate (MTX) treatment duration and dosage with the TST induration diameter of the TST reaction among rheumatoid arthritis (RA) patients.

**Method:**

We retrospectively analyzed consecutive cases of RA patients who were TNF-i therapy candidates. TST measurements, prednisone and methotrexate dosages, and treatment durations were recorded. A control group was randomly selected from healthy subjects. We compared TST reaction size between the following three groups: RA patients with current prednisone treatment, RA prednisone naïve patients, and healthy individuals.

**Results:**

Our study sample comprised 43 RA patients with prednisone treatment, 22 prednisone naïve patients, and 195 healthy subjects. There was no significant difference in mean TST between the groups (5.3±6.6, 7.8±6.2, and 7.6±7.0, respectively, p=0.149). No correlation was noted between TST size and prednisone u-y (r=0.229, p=0.140) or methotrexate u-y in patients with and without prednisone therapy (r=0.219, p=0.158; and r=−0.293, p=0.186, respectively).

**Conclusions:**

Our results show that the TST reaction size among RA patients may not be affected by prednisone therapy. In addition, the TST reaction of RA patients may present similarly to that of healthy individuals. Therefore, we suggest that the criterion of a TST reaction of 5 mm to define latent TB infection in our population should be reevaluated.

## 1. Introduction

With almost 8 million new tuberculosis (TB) cases reported annually, TB morbidity and mortality remain major problems in the world. Although the human immune response is highly effective at controlling the initial infection with* Mycobacterium tuberculosis* [1], in some individuals, all viable organisms may not be eliminated, which leads to a latent tuberculosis infection (LTBI). People with LTBI are asymptomatic, but they harbor Mycobacterium organisms that can be reactivated and cause disease under certain circumstances associated with known risk factors.

It is generally accepted that patients with systemic inflammatory diseases are susceptible to TB infection due to the suppression of their immune systems that can be caused by steroid or disease modifying antirheumatic drug (DMARD) treatment or by the presence of the disease itself. Patients with rheumatoid arthritis (RA) were found to have an increased incidence of TB infection in several studies, including a large observational cohort study from Japan that reported a 3.2-fold increased risk of TB in RA patients who were treated with the standard therapy of DMARDs [2]. Although the introduction of tumor necrosis factor alpha inhibitors (TNF-i) for the treatment of RA represents a significant advance in this area, the therapy is associated with an increase in the incidence of active TB disease among the population of RA patients [3].

Until about 10 years ago, the tuberculin skin test (TST) was the only test available to detect LTBI. However, its accuracy among certain patient groups is questionable. According to the Centers for Disease Control and Prevention guidelines, an induration of ≥ 5 mm is classified as positive in the following groups: patients with HIV infection, patients who had recent close contact with TB infected people, patients who have undergone organ transplants or who are receiving immunosuppression therapy (e.g., TNF-i, methotrexate, prednisone, and cyclosporine), and patients whose chest radiographs show fibrotic changes consistent with previous TB [4]. Although this guideline assigns patients with RA who are being treated with immunosuppressive drugs to the group of patients who are at high risk of developing active TB, it defines neither the dose nor the duration for immunosuppressive treatments, and it only mentions high-dose treatments for patients treated with steroids.

Several factors have been cited as confounders of the TST skin test. Test interpretation is difficult in patients who received Bacille Chalmette-Guerin vaccine or who were exposed to nontuberculous mycobacteria. In patients with rheumatic disease whose immune responses have been compromised, TST may also be misinterpreted. Indeed, among patients with rheumatic disease, test efficacy suffers from low positive and negative predictive values [4, 5].

The aim of the study was to assess the correlation between prednisone and methotrexate (MTX) treatment duration and dosage with the size of the TST reaction among RA patients. In that respect, the size of the TST reaction was compared among RA patients with and without prednisone therapy. In addition, TST reaction size was compared between healthy subjects (HS) and RA patients who were treated with immunosuppressive treatments.

Because the main objective of this study was to compare the distributions of the TST results, assessments of the results in terms of “positive” or “negative” were not done.

## 2. Patients and Methods

This retrospective study was performed in the Rheumatology Unit in collaboration with the Pulmonology Department at Barzilai Medical Center, Israel. The TST was performed according to the Mantoux method by injecting 5 tuberculin units (TU) of purified protein derivative into the inner surface of the forearm and then measuring the maximal size of the induration after 72 hours, as recommended [6]. The study was performed in a single center and all TST measurements were done by a single, highly trained, observer. Anergy was excluded by repeating the TST within two weeks for all subjects whose initial TST result was 0 mm. The second result was considered definitive. Information for the socio-demographic and TB screening questionnaires was obtained from medical charts, and current medical history and treatment were recorded. Overall exclusion criteria were active TB, known history of active TB, and recent immigrant (less than 5 years in Israel). In addition, consecutive cases of RA patients who were candidates for TNF-i therapy were retrospectively reviewed. TST measurements and prednisone and methotrexate doses and treatment durations were recorded. Active tuberculosis (TB) was excluded by chest X-ray and patient history. A control group was randomly selected from healthy individuals who had undergone a TST at the pulmonology clinic of our institution.

We compared the results of the mean size of the TST reaction between the following groups: RA patients who were undergoing prednisone treatment at the time of the TST, RA patients without histories of prednisone treatment, and healthy individuals. A following subanalysis was conducted to include MTX treatments among RA patients. A one-way ANOVA was used to compare the differences between the groups. We introduced the score of unit-year (**u-y**) of the treatment with regard to mean dose of medication and the number years that the medication was being taken. We then calculated** u-y** scores for prednisone and methotrexate by dividing the dosage of the medication by its minimal unit (5 mg/day and 2.5 mg/week, respectively) and then multiplying the result by the number of treatment years. A correlation between these scores and the size of the TST reaction was assessed using Pearson's correlation coefficient (r). Values of p < 0.05 were considered significant.

## 3. Results

Our study sample comprised of 43 (mean age 57.7 ± 13.1 years, 86% female) RA patients with prednisone treatment, 22 (mean age 59.9 ± 10.8 years, 73% female) prednisone naïve patients, and 195 (mean age 51.5 ± 10.9 years, 67% female) healthy subjects (HS). The mean ages between the RA patient groups and the HS group differed significantly, and therefore, a significantly higher incidence of systemic hypertension was found in the RA groups. There was no difference in other diseases between the groups. The baseline characteristics of RA patients and HS are shown in Table 1.

**Figure 1:**
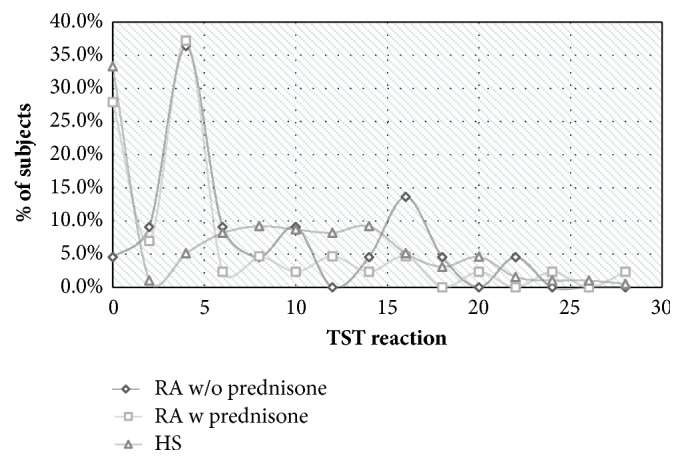
TST reactions in RA patients treated with prednisone, in those not treated with prednisone and in controls. TST = tuberculin skin test; RA = rheumatoid arthritis; HS = healthy subjects.

We did not find any significant difference in the mean TST values between the three groups. In RA patients with prednisone treatment, mean TST size was 5.3 mm ± 6.6 mm; in the group of RA patients without prednisone treatment, mean TST size was 7.8 mm ± 6.2 mm; and in the group of HS mean TST size was 7.6 mm ± 7.0 mm, p = 0.149 (Table 2 and Figure 1).

**Figure 2:**
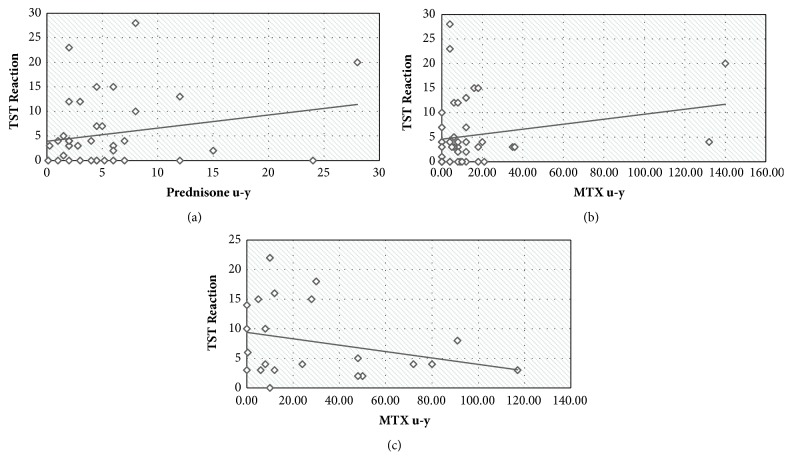
Correlation between TST size and (a) prednisone u-y in RA patients with prednisone therapy, (b) MTX u-y in RA patients with prednisone therapy, and (c) MTX u-y in RA patients without prednisone therapy. TST = tuberculin skin test; u-y = unit years; RA = rheumatoid arthritis; MTX = methotrexate.

No correlation was found between TST size and prednisone [mean** u-y** = 5.2 ± 5.6, (r = 0.229, p = 0.140)] and between TST size and methotrexate** u-y** in patients with prednisone therapy [mean u-y = 14.4 ± 28.2 (r = 0.219, p = 0.158)] or without prednisone therapy [mean u-y = 30.7 ± 32.4 (r = −0.293, p = 0.186)] (Figure 2).

## 4. Discussion

An accurate diagnosis of LTBI in patients before starting TNF-i treatment and subsequent prophylactic anti-TB treatment is critical to prevent TB reactivation. The currently accepted screening strategy, based on TST, chest x-rays, and the data collected from the risk stratification questionnaire, has significantly reduced the rate of TB reactivation under TNF-i therapy [7]. Despite this success, the correct approach to diagnosing LTBI is still being debated. The main issue surrounds the interpretation of TST results among patients with inflammatory arthritis [8]. In RA patients being treated with immunosuppressive drugs, misinterpretation can lead to false negative test results with the potential to cause serious harm to patients. To avoid false negative results among patients, the recommended cut-off of induration size is 5 mm.

The results of our study, which did not find attenuation in TST results among RA patients in comparison with HS patients, contrasted those of previous studies that showed an attenuated response [9, 10] but were in agreement with the results of several other studies [3, 11, 12]. The observed discrepancies are probably due to differences between certain features of the countries where the studies were performed. For example, although in developing countries, the TST reaction may be attenuated as a result of poor socioeconomic conditions and of additional diseases status, in developed countries, these effects are diminished or absent.

Reducing the cutoff value for a positive TST reaction to 5 mm among RA patients who are being treated with immunosuppressive drugs may lead to the detection of more cases of LTBI. Although this increase would come at the cost of a corresponding increase in the proportion of false positive results, the reduced cut-off value is justified if the attenuating effect that immunosuppressive drugs have on TST size reaction is strongly proved. High doses of prednisone (more than 15 mg/d) are known to have an attenuating effect on TST size about which all studies are in agreement. However, the efficacy of the long-term treatment of RA patients with low doses of prednisone (less than 10 mg) has not been definitively determined [10, 11, 13].

In our study, the use of low-dose prednisone was not associated with reductions in the TST induration size in RA patients in comparison to HS patients, even when treatment had been ongoing for several years. Likewise, MTX had no effect on TST results as in a previous study [11]. Therefore, considering the results of our study together with those of previous works, lowering the cut-off of induration size for the TST reaction in RA patients may be warranted in locations with high prevalences of TB infection. In other places, where the prevalence of TB infection is low, such a decrease may lead to only a limited reduction in false negative results. In addition, it could also result in an increase in false positive TST results and subsequently in the provision of unnecessary treatment.

Our study has several limitations. First, the small number of subjects (n = 260) may reduce the chances of obtaining statistically significant results. Second, we did not analyze the correlation between TST results and disease activity, and we could not provide BCG status for the study participants. However, as our study participants may had received BCG vaccination only during early childhood, the mean age of the participants suggests their previous vaccination should not influence TST, as BCG vaccination protection declines with time and generally lasts for up to 10 years [14]. Third, although TST exam is subjective and intraobserver variation can occur, golden standard of latent tuberculosis diagnosis was not available to us as QuantiFERON test or Golden T-Spot test is not routinely performed in our country. Finally, the mean age of our group of patients in the study was significantly higher than that of the healthy controls, a scenario that may lead to lower diameters of TST in the patient group compared with the healthy controls.

In conclusion, the evidence presented here suggests that adherence to the accepted TST-based recommendations for the diagnosis of LTBI based on the cut-off for TST reaction size of 5 mm may lead to overestimates of the size of the effect in the form of excessively high numbers of positive results that, in turn, cause LTBI to be overdiagnosed. Our data elicit the suggestion that the algorithms used to test for LTBI are revised, particularly for nonendemic populations. In fact, for such populations, the substitution of the traditional TST with newer diagnostic tools like the QuantiFERON assay may be warranted. Larger studies are needed to verify our results.

## Figures and Tables

**Table 1:** Baseline characteristics of study participants.

**Variable**	**RA Patients (n = 65)**	**Controls (n = 195)**	**P value**
**Age (yrs)**	58.4 ± 12.3	51.5 ± 10.9	<0.001
**Female (**%**)**	53 (82)	131 (67)	0.028
**Comorbidities**			
**Systemic hypertension (**%**)**	38 (59)	64 (33)	<0.001
**Diabetes mellitus (**%**)**	10 (15)	22 (11)	0.383
**Cardiovascular disease (**%**)**	3 (5)	16 (8)	0.420
**Chronic lung disease (**%**)**	2 (3)	14 (7)	0.372
**Chronic renal insufficiency (**%**)**	3 (5)	9 (5)	0.999
**Liver disease (**%**)**	1 (2)	9 (5)	0.459
**History of malignancy (**%**)**	2 (3)	5 (3)	0.999

**Table 2:** Mean and median TST sizes in RA patients treated with prednisone and RA patients not treated with prednisone and controls.

**TST size (mm)**	**RA with prednisone therapy (n = 43)**	**RA without prednisone therapy (n = 22)**	**Controls (n = 195)**	**P value**
**Mean ± SD**	5.3 ± 6.6	7.8 ± 6.2	7.6 ± 7.0	0.149
**Median**	3.0	4.5	7.0	
**Range**	[0, 28]	[0, 22]	[0, 27]	

## Data Availability

The data used to support the findings of this study are included within the article.
